# Discovery of Hippo signaling as a regulator of CSPG4 expression and as a therapeutic target for *Clostridioides difficile* disease

**DOI:** 10.1371/journal.ppat.1011272

**Published:** 2023-03-27

**Authors:** Jason L. Larabee, D. Annie Doyle, Ummey Khalecha Bintha Ahmed, Tyler M. Shadid, Rachel R. Sharp, Kenneth L. Jones, Young Mi Kim, Shibo Li, Jimmy D. Ballard

**Affiliations:** 1 Department of Microbiology and Immunology, The University of Oklahoma Health Sciences Center, Oklahoma City, Oklahoma, United States of America; 2 Laboratory for Molecular Biology and Cytometry Research, Harold Hamm Diabetes Center, Oklahoma City, Oklahoma, United States of America; 3 Department of Cell Biology, The University of Oklahoma Health Sciences Center, Oklahoma City, Oklahoma, United States of America; 4 Department of Pediatrics, The University of Oklahoma Health Sciences Center, Oklahoma City, Oklahoma, United States of America; University of Pittsburgh School of Medicine, UNITED STATES

## Abstract

The signaling pathways and networks regulating expression of chondroitin sulfate proteoglycan 4 (CSPG4), a cancer-related protein that serves as a receptor for *Clostridiodes difficile* TcdB, are poorly defined. In this study, TcdB-resistant/CSPG4-negative HeLa cells were generated by exposure to increasing concentrations of the toxin. The cells that emerged (HeLa R5) lost expression of *CSPG4* mRNA and were resistant to binding by TcdB. mRNA expression profiles paired with integrated pathway analysis correlated changes in the Hippo and estrogen signaling pathways with a CSPG4 decrease in HeLa R5 cells. Both signaling pathways altered CSPG4 expression when modulated chemically or through CRISPR-mediated deletion of key transcriptional regulators in the Hippo pathway. Based on the in vitro findings, we predicted and experimentally confirmed that a Hippo pathway inactivating drug (XMU-MP-1) provides protection from *C*. *difficile* disease in a mouse model. These results provide insights into key regulators of CSPG4 expression and identify a therapeutic for *C*. *difficile* disease.

## Author summary

CSPG4 is a cancer-associated protein and a receptor for *C*. *difficile* TcdB. Though CSPG4 is important in both cancer and infection, little is known about the signaling networks regulating its expression. In this study, estrogen and Hippo signaling were found to independently impact *CSPG4* transcription and the detectable levels of CSPG4 in cells. Based on this information, we used a small molecule inhibitor of Hippo signaling to block CSPG4 expression during *C*. *difficile* infection in a rodent model. Doing so protected against *C*. *difficile* disease by reducing tissue pathologies, inflammation, and weight loss following infection. Overall, results from this study provides a significant advance in both understanding how CSPG4 expression is regulated and the prospect of targeting CSPG4 regulators to reduce toxin effects during *C*. *difficile* disease.

## Introduction

Chondroitin sulfate proteoglycan 4 (CSPG4) is a type 1 transmembrane cell surface protein glycosylated at multiple sites and modified with up to three chondroitin sulfate glycosaminoglycans [1]. CSPG4 (also commonly referred to as NG2) interacts with the extracellular matrix and inordinate expression of CSPG4 is associated with cancers such as glioblastoma, melanoma and carcinoma [2–6]. Transcriptional regulation of *CSPG4* is influenced through differential methylation of a 1585 bp 5’ UTR promoter region of *CSPG4* [7], and a 1450 bp intronic region of the *CSPG4* gene functions as an enhancer that responds to Sox10 and bHLH transcriptional regulators [8]. Other transcription factors shown to regulate *CSPG4* include Sp1, Pax3, and Egr-1 [9–11]. Cytokines and hypoxia influence CSPG4 levels [12–14], but signaling networks through which these factors activate or repress expression of CSPG4 are poorly defined. Indeed, little is known about the signaling networks that influence expression of CSPG4.

*Clostridioides difficile* TcdB exploits CSPG4 as a receptor for binding to and entering target cells during infection [15]. Following receptor binding, TcdB enters cells where it inactivates small GTPases via glucosylation [16]. At least eight variants of TcdB are produced within two major clades of *C*. *difficile* and collectively these different forms of TcdB engage up to four different cell-surface receptors: Nectin-3 [17], Frizzled (FZD) [18], tissue factor pathway inhibitor (TFPI) [19] and CSPG4 [15]. Though CSPG4 is a common receptor for many of the variants of TcdB, the overall receptor profiles varies between the two major forms of TcdB (TcdB1 and TcdB2), which are produced by distinct clinically-relevant strains of *C*. *difficile* [20]. TcdB1, but not TcdB2, interacts with FZD [18]. Conversely, TcdB2, but not TcdB1, interacts with TFPI [19]. Both TcdB variants bind to CPSG4, but TcdB2 appears to rely more on CSPG4 for cellular intoxication than does TcdB1. For example, CSPG4^-/-^ HeLa cells are greater than 10-fold more resistant to TcdB2 than TcdB1 [21]. As reported by Chen and colleagues, in comparison to wild-type mice, intestinal damage is significantly reduced when CSPG4 KO mice are infected with a strain of *C*. *difficile* expressing TcdB2 [22]. As such, investigations into CSPG4, including identification of factors regulating its expression, is particularly key to elucidating the overall role of TcdB2 in *C*. *difficile* disease.

To identify putative CSPG4-regulatory signaling pathways, HeLa cells were treated with increasing concentrations of TcdB2 until TcdB-resistant/CSPG4-negative cells emerged. These CSPG4-negative cells had an intact CSPG4 gene but exhibited chromosomal rearrangements that clearly differentiated them from parental cells. RNA-Seq data paired with bioinformatic analysis identified signaling networks altered in the TcdB2-resistant cells, which led to the discovery of estrogen and the Hippo signaling pathway as regulators of CSPG4 expression. Capitalizing on these findings, Hippo signaling was modulated in vivo with a chemical inhibitor and was found to protect mice from weight loss and tissue pathologies associated with *C*. *difficile* infection.

## Results

### Generation of TcdB2-Resistant HeLa Cells

**Fig 1:**
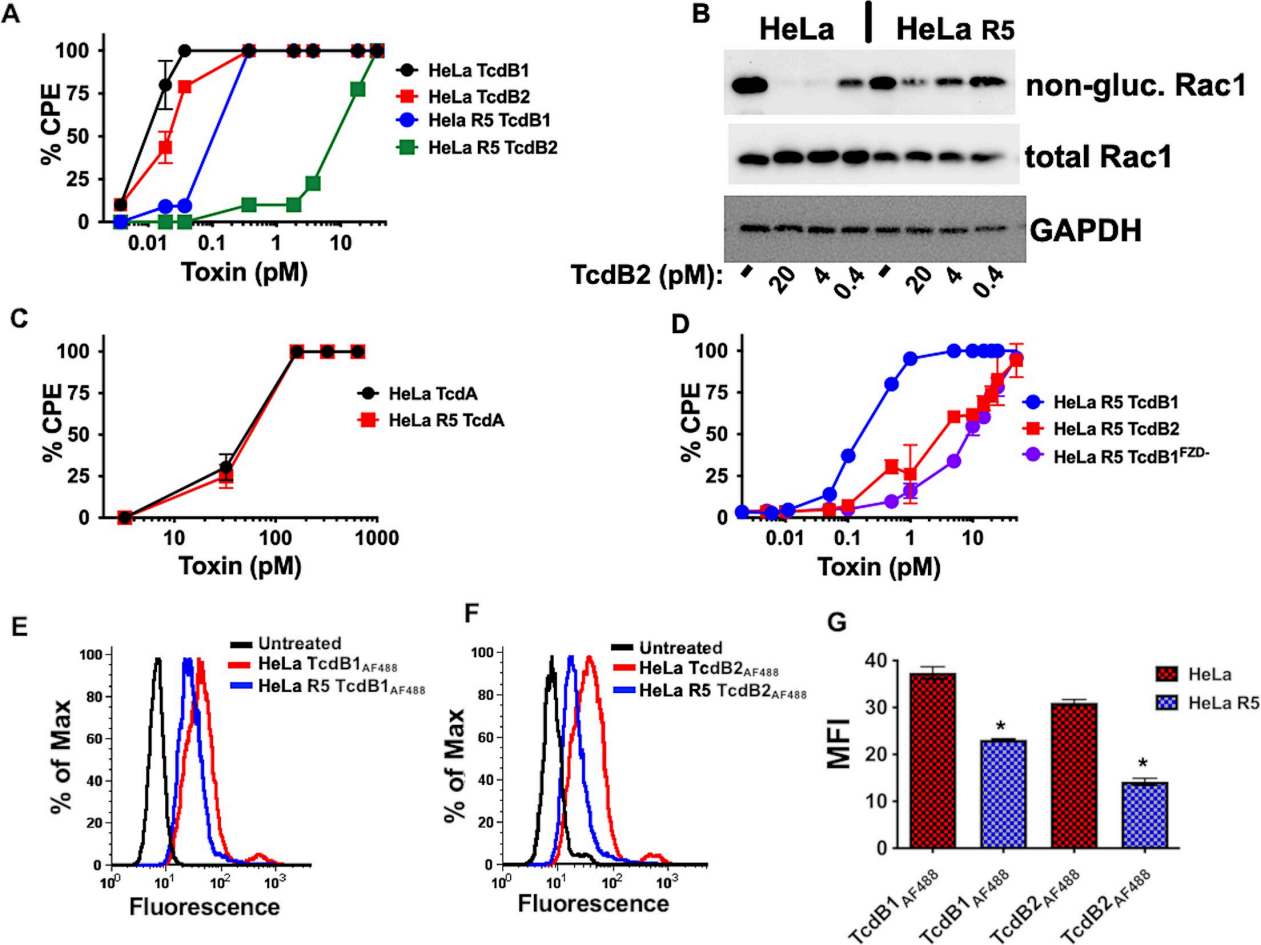
Toxin effects in HeLa and HeLa R5 cells. (*A*) Cytotoxicity assay quantifying cytopathic effects (CPE) in parental HeLa cells and HeLa R5 cells following 24 h treatment with either TcdB1 or TcdB2. (*B*) Immunoblot analysis of Rac1 glucosylation in HeLa and HeLa R5 cells. Cells were treated for 5 h with TcdB2 and protein lysates were collected for immunoblot analysis. Individual immunoblots were probed using antibodies recognizing non-glucosylated Rac1, total Rac1, or GAPDH. (*C*) Cytotoxicity assay quantifying CPE in HeLa and HeLa R5 cells following 24 h treatment with TcdA. (*D*) Cytotoxicity assay quantifying CPE in HeLa R5 cells following 24 h treatment with either TcdB1, TcdB2, or TcdB1^FZD-^. All CPE data are presented as the mean ± standard deviation for samples examined in triplicate. *(E-G)* TcdB binding to HeLa and HeLa R5 cells was measured by flow cytometry. Cells were exposed to 100 nM of TcdB1_AF488_ or 100 nM of TcdB2_AF488_ for 30 min on ice before a series of washes and assessment by flow cytometry. (*E*,*F*) Histograms comparing fluorescent signal from HeLa and HeLa R5 cells exposed to TcdB1_AF488_ or TcdB2_AF488_. (*G*) Median fluorescent intensity (MFI) from flow cytometry data in three experiments ± S.D. * *p* < 0.05 determined by Student’s t-test.

HeLa cells were exposed to 0.02 pM of TcdB2, with stepwise increases in toxin concentration up to 20 pM over a course of approximately one month. TcdB2-resistant cells emerged from this treatment and were termed HeLa R5. When compared to parental HeLa cells, HeLa R5 cells were ~400-fold less sensitive to TcdB2 and ~10 fold less sensitive to TcdB1 (*Fig 1A*). Glucosylation of Rac1 (an intracellular target of TcdB), was compared between the parental and HeLa R5 cells treated with TcdB2. As shown in *Fig 1B*, antibody recognition of non-glucosylated Rac1 was reduced in TcdB2-treated HeLa cells but not in HeLa R5 cells.

TcdA, which glucosylates a similar subset of small GTPases but enters cells via mechanisms different from TcdB, was used to further assess whether HeLa R5 resistance was due to changes in substrate modification or cell entry. As shown in *Fig 1C*, HeLa and HeLa R5 cells were equally sensitive to TcdA, suggesting the resistance was not due to changes in substrate or cosubstrate utilization.

The data shown in *Fig 1A–1C* supported the notion that HeLa R5 cells had been altered in a way that primarily reduced sensitivity to TcdB2, but had only a minimal effect on TcdB1 and no detectable effect related to TcdA. This sensitivity profile correlated with TcdB2’s heightened use of CSPG4, and led us to predict that modifying TcdB1 to have a similar dependence on CPSG4 would result in identical HeLa R5 cytotoxicity profiles for both forms of this toxin. To test this, cytotoxicity was measured in HeLa R5 cells using a TcdB1 mutant (TcdB1^FZD-^) with the amino acid residues necessary for the FZD binding (D1501, Y1509, N1511, F1597) converted to glycines. Loss of FZD binding by this mutant was confirmed by microscale thermophoresis (*S1 Fig*). In line with our prediction, TcdB1^FZD-^ caused cytopathic effects on HeLa R5 cells almost identical to that of TcdB2 (*Fig 1D*).

Based on the initial results, we suspected TcdB2 cell binding, and to a lesser extent TcdB1 cell binding, would be reduced on HeLa R5 cells. To examine this possibility, cell binding by Alexa Fluor 488-labeled TcdB1 and TcdB2 was examined by flow cytometry. As shown in *Fig 1E–1G*, cellular interactions of both TcdB1_AF488_ and TcdB2_AF488_ were reduced in HeLa R5 cells compared to parental HeLa cells. TcdB2_AF488_ binding to HeLa R5 cells was reduced approximately 60% compared to parental HeLa cells, whereas the binding of TcdB1_AF488_ in Hela R5 cells was reduced approximately 30%.

### Loss of CSPG4 in HeLa R5 Cells

**Fig 2:**
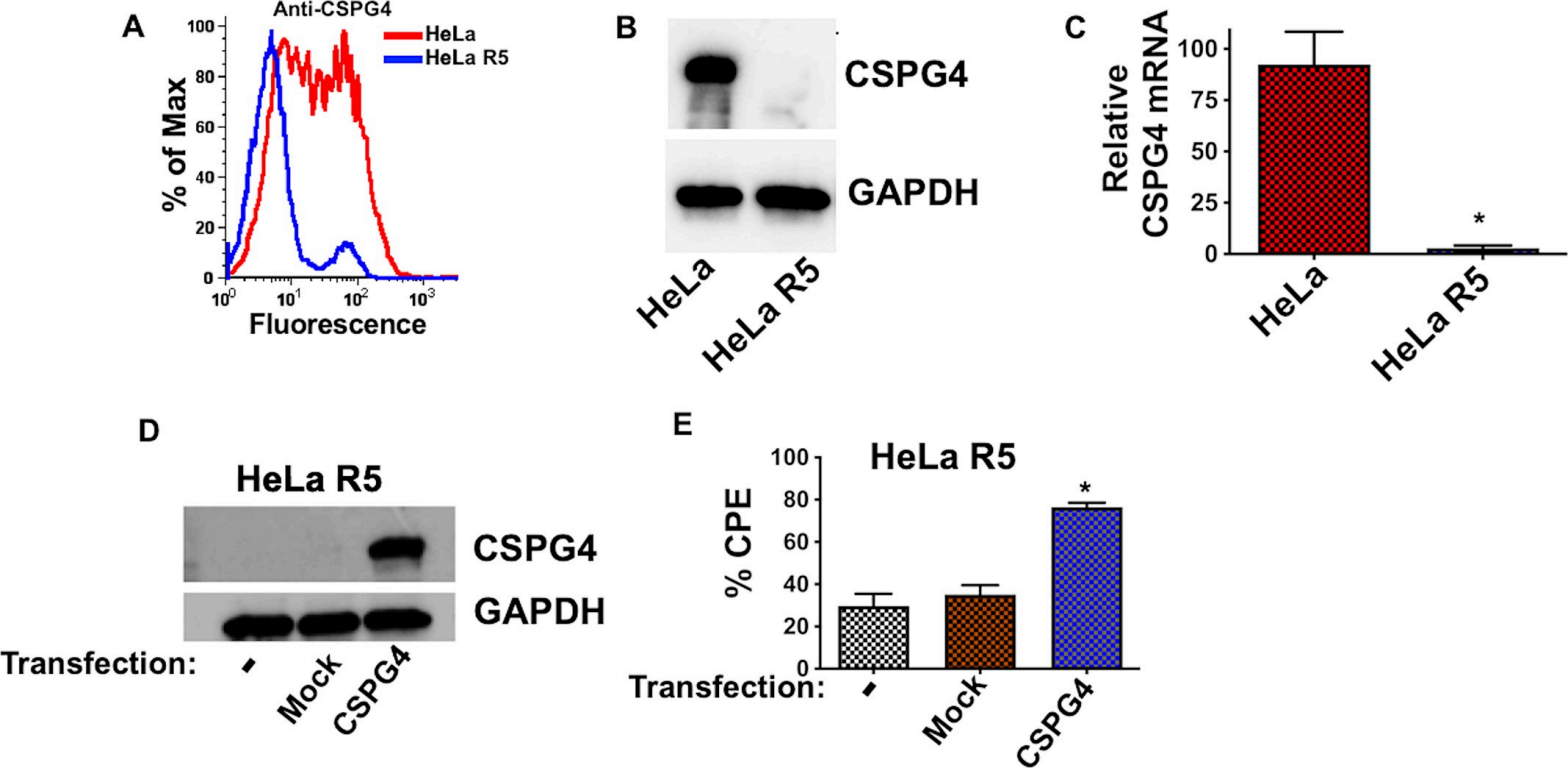
CSPG4 levels in HeLa and HeLa R5 cells. (*A*) Flow cytometry analysis of HeLa and HeLa R5 cells stained with antibodies against CSPG4. *(B)* Immunoblot analysis of CSPG4 and GAPDH levels using protein lysates from HeLa and HeLa R5 cells. *(C)* RNA was extracted from HeLa and HeLa R5 cells, and RT-qPCR was used to quantify *CSPG4* transcripts. RT-qPCR data are presented as mean (n  =  3) ± S.D. (*D*) Immunoblot analysis of CSPG4 in HeLa R5 and HeLa R5 cells transfected with pEF6-CSPG4-myc-his. (*E*) Comparison of TcdB2 CPE in HeLa R5 and HeLa R5 (pEF6-CSPG4-myc-his) after treatment with 4 pM of toxin for 24 h. * *p* < 0.05 determined by Student’s t-test.

The results shown in *Fig 1* suggested CSPG4 levels were reduced in HeLa R5 cells, which led us to compare CSPG4 levels between HeLa and HeLa R5 cells. As shown in *Fig 2A and 2B*, relative CSPG4 levels between HeLa and HeLa R5 cells were measured by flow cytometry and immunoblot. Results from both approaches indicated CSPG4 was below levels of detection in HeLa R5 cells. In line with this, and as shown in *Fig 2C*, analysis of *CSPG4* mRNA levels indicated transcripts were >75 fold lower in HeLa R5 cells compared to the parental HeLa cells. We also examined the recently identified TcdB2 receptor TFPI [19] and found that TFPI was not decreased in HeLa R5 cells compared to the parental HeLa cells (immunoblot in *S2 Fig*).

To directly implicate loss of CSPG4 with HeLa R5 resistance, CSPG4 was ectopically expressed in HeLa R5 cells and sensitivity to TcdB2 was measured in these cells. Transfection of HeLa R5 cells with a CSPG4 expression plasmid resulted in detectable levels of CSPG4, as determined by immunoblot (*Fig 2D*). Moreover, when CSPG4 was expressed in HeLa R5 cells, sensitivity to TcdB2 was recovered (*Fig 2E*).

To determine the stability of HeLa R5 resistance to TcdB2, the cells were passaged in the absence of TcdB2 for 16 weeks and then analyzed for *CSPG4* transcript levels. HeLa R5 cells did not revert to expressing parental cell levels of CSPG4 after passage for 16 weeks (*S3 Fig*), suggesting resistance to TcdB2 was stable and did not require continual exposure to the toxin.

### Karyotype analysis and characterization of the CSPG4 Gene in HeLa R5 Cells

**Fig 3:**
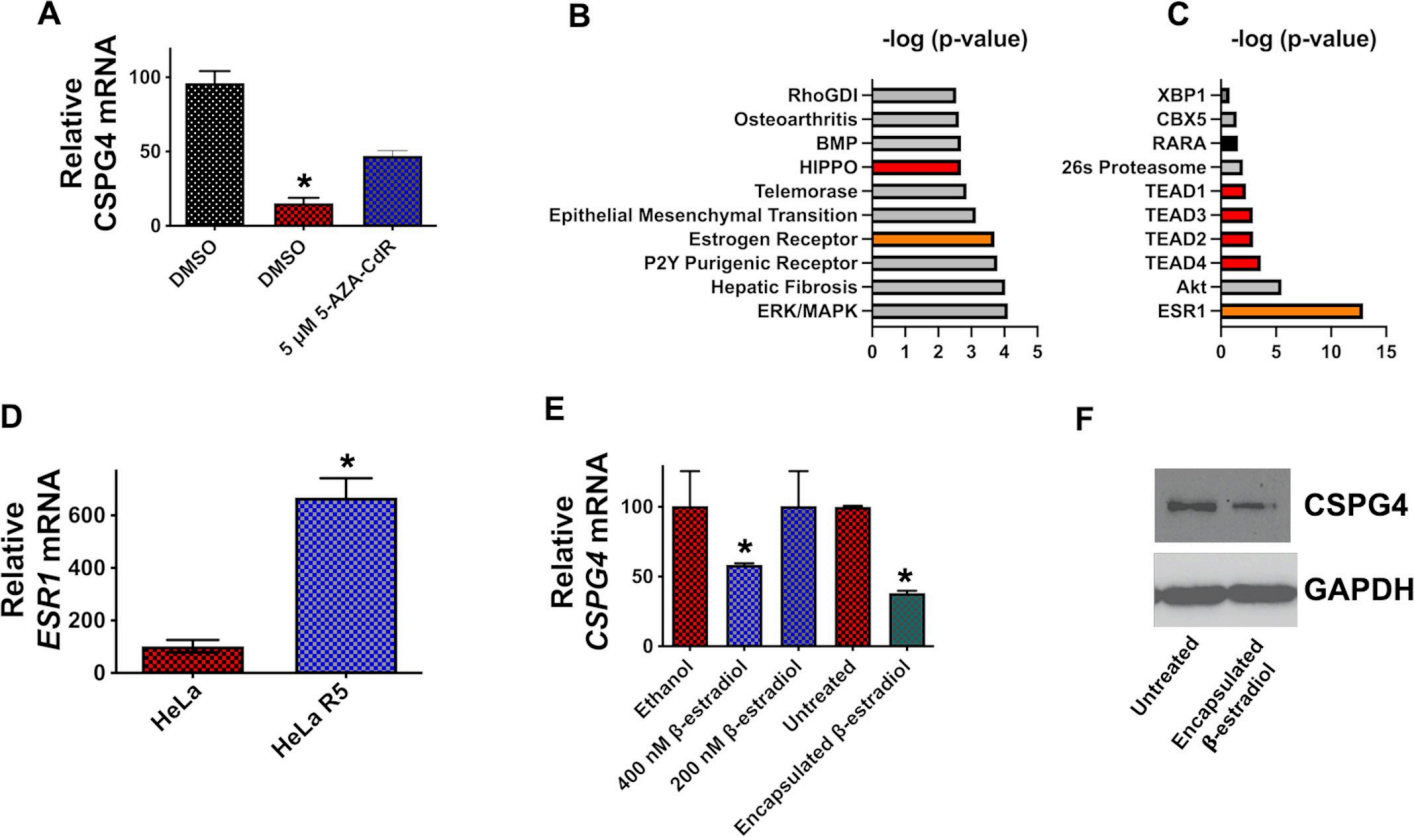
Regulation of CSPG4 expression. (*A*) HeLa R5 cells were exposed to 5-AZA-CdR for 48 h and then RT-qPCR was used to quantify CSPG4 transcripts. RT-qPCR data are presented as mean (n  =  3) ± S.D. (*B*) The top statistically significant canonical pathways correlating (positive z score) with HeLa R5 cells. (*C*) Upstream regulators predicted to be activated in HeLa R5 cells based on integrated pathway analysis. (*D*) Comparison of *ESR1* transcripts between HeLa and HeLa R5 cells presented as the mean (n  =  3) ± S.D. (*E*) HeLa cells were cultured in 0.5% charcoal stripped FBS and phenol red free media for 2 h prior to a 24 h exposure to non-encapsulated ß-estradiol or encapsulated ß-estradiol. RT-qPCR was then utilized to quantify CSPG4 transcripts exhibited as mean (n  =  3) ± S.D. (*F*) HeLa cells were exposed to encapsulated ß-estradiol for 24 h and then an immunoblot was performed on the resulting protein extracts. *p < 0.05 determined by Student’s t-test.

The next series of experiments sought to identify possible explanations for loss of CSPG4 expression in HeLa R5 cells. Karyotype analysis indicated both of these cell lines possess similar chromosome structural abnormalities as typical HeLa cells but the numbers of chromosomes differed in HeLa R5 cells compared to parental HeLa cells (*S4 Fig*). The two cell lines were also subjected to whole-genome sequencing. Although the extensive chromosomal changes identified in the karyotype analysis limited the usefulness of direct comparisons between the genome sequences, specific DNA analysis of the *CSPG4* gene did not identify sequence changes that could impact transcription of this gene. Previous studies have shown *CSPG4* expression is reduced through DNA methylation [23]; therefore, we also used treatment with a DNA methylation inhibitor (5-AZA-CdR) to confirm that this gene is transcriptionally functional in HeLa R5 cells. As shown in *Fig 3A*, *CSPG4* transcript levels increased in HeLa R5 cells treated with a DNA methylation inhibitor (5-AZA-CdR). A corresponding increase in sensitivity to TcdB2 was also observed in HeLa R5 cells following 5-AZA-CdR treatment (*S5 Fig*). These findings indicate loss of CSPG4 in HeLa R5 cells is not due to mutations in the *CSPG4* gene or its capacity to be transcribed.

### Identification of CSPG4-Related signaling networks altered in HeLa R5 Cells

To elucidate how the genetic changes in HeLa R5 cells lead to CSPG4 repression, whole-transcriptome sequencing (RNA-seq) datasets were generated from HeLa and HeLa R5 cells and examined by Ingenuity Pathways Analysis (IPA) to find signaling networks that correlated with a loss in CSPG4. As shown in *Fig 3B*, the top statistically significant canonical pathways are presented that correlate (positive z score) with HeLa R5 cells. The upstream regulators predicted to be activated in HeLa R5 cells are shown in *Fig 3C*. Estrogen (orange shading in *Fig 3B and 3C*) and Hippo (red shading in *Fig 3B and 3C*) signaling emerged as two key pathways with strong correlations to HeLa R5 cells.

In addition to IPA analyses highlighting estrogen signaling, RT-qPCR revealed that HeLa R5 cells expressed higher levels of *ESR1* than HeLa cells (*Fig 3D*). Taken together, these data led us to investigate whether estrogen signaling impacts the expression of CSPG4 in HeLa cells. As shown in *Fig 3E and 3F*, when HeLa cells were exposed to encapsulated ß-estradiol, CSPG4 at both the mRNA and protein level (bands quantified in *S6 Fig*) were found to be decreased. This experiment was also performed under conditions (0.5% charcoal stripped FBS and phenol red free media) where ESR1 ligands were depleted from the media. Results of this experiment demonstrated that encapsulated ß-estradiol as well as non-encapsulated ß-estradiol reduce levels of the *CPSG4* transcript (*Fig 3E*). In addition, these results indicate HeLa R5 cells are useful for discovering signaling networks regulating CSPG4 levels.

### The role of Hippo Signaling in CSPG4 regulation

**Fig 4:**
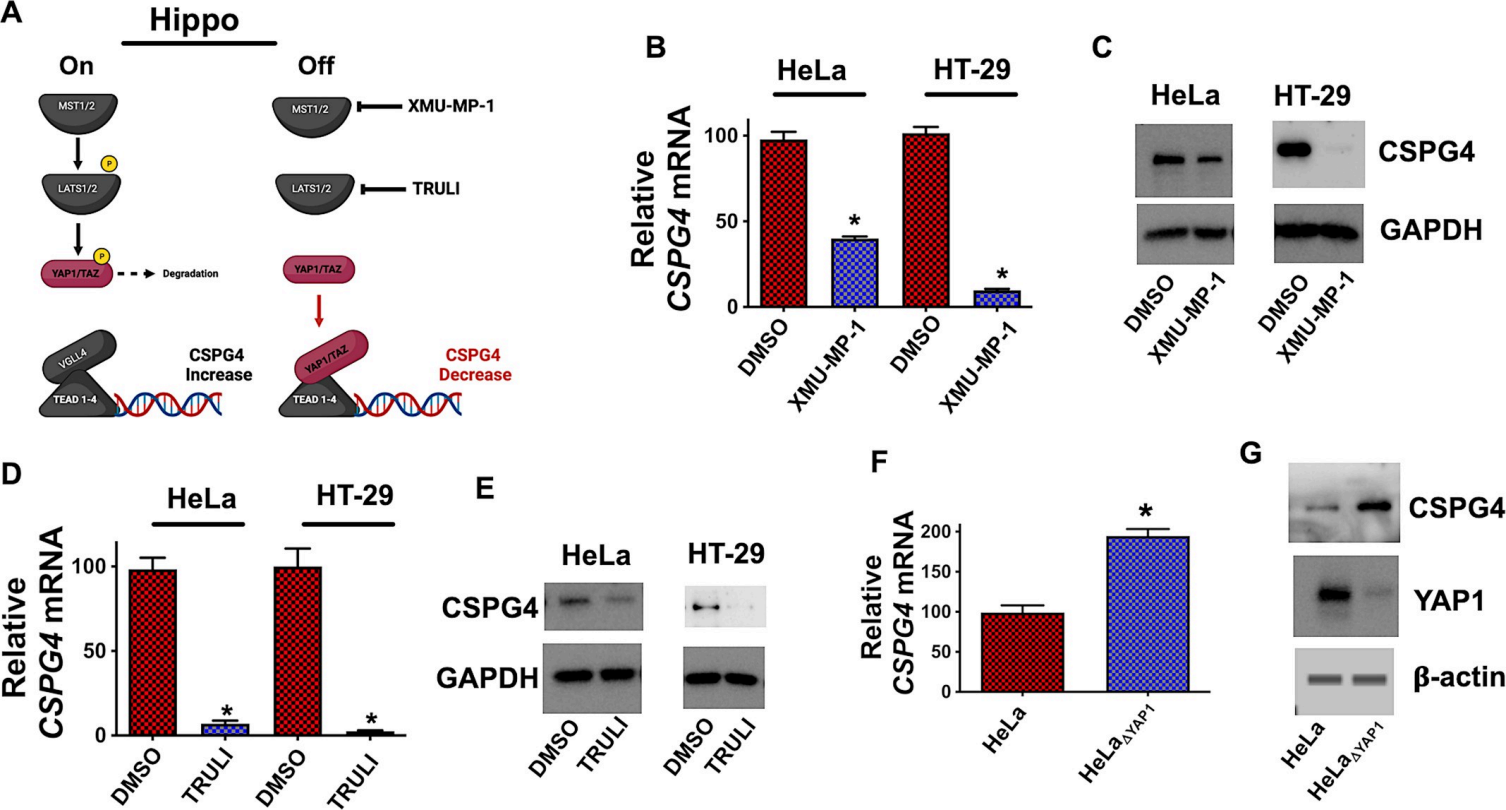
Hippo signaling regulates CSPG4 levels. (*A*) Depiction of the Hippo signaling pathway and the effect of inhibition by XMU-MP-1 and TRULI. (*B*) *CSPG4* transcript levels in HeLa and HT-29 cells treated with 3 μM XMU-MP-1 for 7 h. *CSPG4* transcript levels are presented as mean (n  =  3) ± S.D. (*C*) Immunoblot detection of CSPG4 from HeLa and HT-29 cells exposed to 6 μM of XMU-MP-1 for 24 h. (*D*) *CSPG4* transcript levels in HeLa and HT-29 cells treated with 30 μM TRULI for 24 h. *CSPG4* transcript levels are presented as mean (n  =  3) ± S.D. *(E)* Immunoblot detection of CSPG4 from HeLa and HT-29 cells exposed to 30 μM of TRULI for 24 h. (*F*) *CSPG4* transcript levels in HeLa and YAP1 knock out cells (HeLa_ΔYAP1_). *CSPG4* transcript levels are presented as mean (n  =  3) ± S.D. (*G*) Immunoblot analyzing YAP1 and CSPG4 levels in HeLa cells and HeLa_ΔYAP1_. *p < 0.05 determined by Student’s t-test.

IPA identified TEAD1-4 as potential upstream regulators (*Fig 3C*) active in HeLa R5 cells. TEA domain (TEAD) proteins are a family of transcription factors that respond to Hippo transcriptional coactivators, Yes-associated protein 1 (YAP1) and Transcriptional coactivator with PDZ-binding motif (TAZ). As illustrated in *Fig 4A*, YAP1/TAZ-mediated gene transcription is repressed when Hippo is in the “on” state. Hippo signaling is initiated when the MST1/2 kinase phosphorylates the LATS1/2 kinase and continues as active LATS1/2 phosphorylates YAP1/TAZ, thereby promoting ubiquitination and degradation of YAP1/TAZ. When Hippo signaling is “off”, MST1/2 and LAT1/2 are inactive and this allows YAP1/TAZ to accumulate and bind TEAD to activate gene transcription. Hippo signaling is deactivated using pharmacological inhibitors of MST1/2 (XMU-MP-1) or LATS1/2 (TRULI) as depicted in *Fig 4A* [24,25]. In the following experiments, these inhibitors were used to determine if Hippo signaling regulates CSPG4.

As shown in *Fig 4B*, inactivation of MST1/2 using XMU-MP-1 resulted in the repression of *CSPG4* transcripts in HeLa cells and in a colonic epithelial cell line (HT-29). Similar levels of CSPG4 repression were observed at the protein level in HeLa and HT-29 cells treated with XMU-MP-1 (*Figs 4C and S6*). Likewise, inactivation of LATS1/2 with TRULI reduced CSPG4 at both the transcript and protein level in HeLa and HT-29 cells (*Figs 4D, 4E* and S6). Using chemical inhibitors to modulate Hippo signaling points to active YAP1 reducing CSPG4 levels; therefore, we predicted the absence of YAP1 should increase the expression of CSPG4. To test this, YAP1 was deleted in HeLa cells using CRISPR/Cas9 gene editing, resulting in the cell line HeLa_ΔYAP1_. After confirming YAP1 deletion by immunoblot (*Fig 4G*), CSPG4 levels were examined at both the transcript and protein level, and CSPG4 was found to be increased in HeLa_ΔYAP1_ cells compared to parental HeLa cells (*Fig 4F and 4G*).

### XMU-MP-1 represses CSPG4 expression in the mouse colon and prevents *C*. *difficile* disease

Experiments next determined if *C*. *difficile* disease could be attenuated by eliminating CSPG4 expression through pharmacological targeting of Hippo signaling with XMU-MP-1. Prior to the in vivo testing, we confirmed that XMU-MP-1 did not inhibit the growth of *C*. *difficile*. As shown in *S1 Table*, *C*. *difficile* achieved similar levels of growth in the presence and absence of XMU-MP-1.

**Fig 5:**
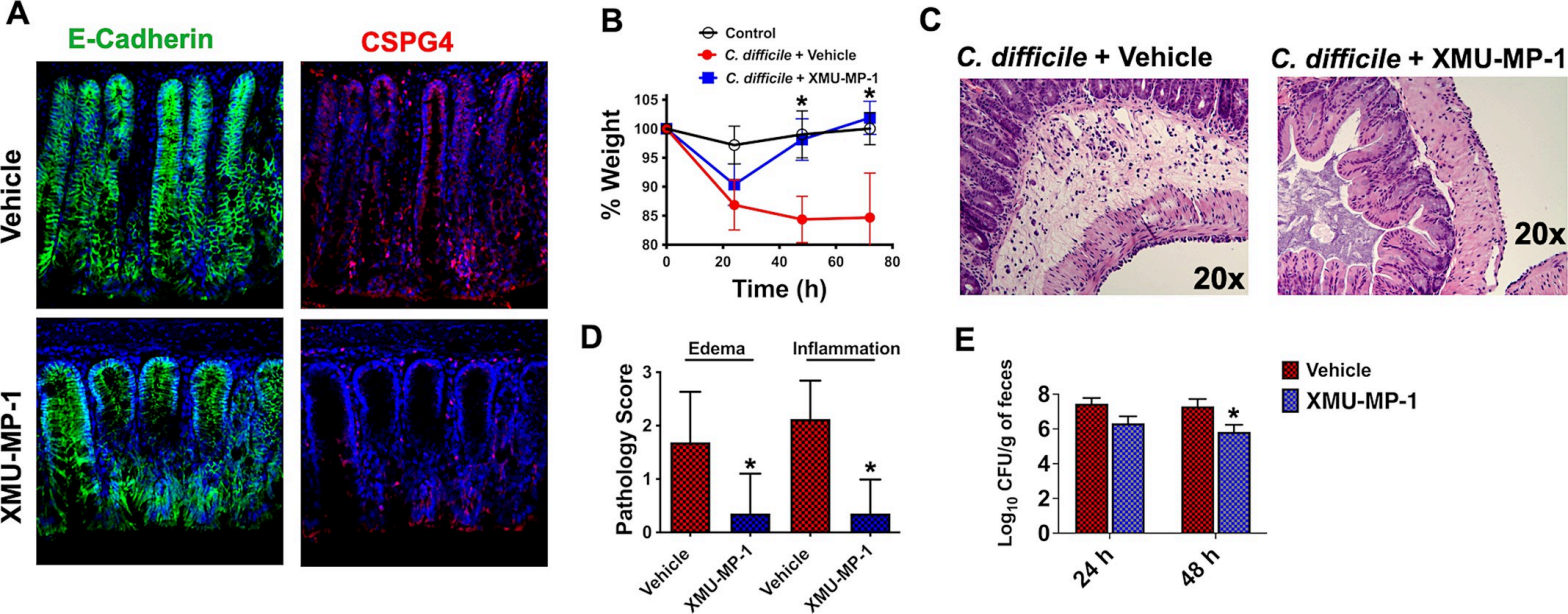
Amelioration of *C*. *difficile* disease in mice treated with XMU-MP-1. Mice were administered intraperitoneal injections of XMU-MP-1 (1 mg/kg) or vehicle daily for two days prior to oral gavage of 10^6^ *C*. *difficile* spores. XMU-MP-1 was administered daily following infection for a total of 5 injections during the course of the experiment. *(A)* Immunofluorescence staining of colon excised from mice 24 h after the last injection with XMU-MP-1 or vehicle. *(B)* Percent change in weight in mice post infection presented as mean (n  =  6) ± S.D. *(C)* Representative H&E images of mouse ceca at 96 h post infection. *(D)* Pathology scores of mouse ceca at 96 h post infection for vehicle (n = 16) ± S.D and XMU-MP-1 (n = 14) ± S.D. *(E)* Levels of *C*. *difficile* in the fecal pellets from mice infected with *C*. *difficile* with and without exposure to XMU-MP-1. *p < 0.05 indicates significance between vehicle and XMU-MP-1 treatment as determined by Student’s t-test.

To determine if XMU-MP-1 could provide a therapeutic benefit during *C*. *difficile* infection, mice were administered 1 mg/kg of XMU-MP-1 daily by intraperitoneal injection starting 48 h prior to *C*. *difficile* infection and continuing through the length of the experiment. In this infection model depicted in *S7 Fig*, mice were sensitized to *C*. *difficile* through a 5 day exposure to the antibiotic cefoperazone [26]. Following this antibiotic exposure and a 2 day recovery, mice underwent oral gavage with 10^6^ spores of a TcdB2 producing *C*. *difficile* (R20291) strain. CSPG4 levels were examined by excising colons from infected mice 24 h after the final XMU-MP-1 injection and then staining for CSPG4 on colonic sections. As shown in *Fig 5A*, a comparison of control mice with XMU-MP-1 treated mice revealed colonic CSPG4 staining was substantially reduced in the mice that received XMU-MP-1, while the colonic marker E-cadherin remained unchanged.

Next, *C*. *difficile* disease was evaluated in mice receiving vehicle or XMU-MP-1. *C*. *difficile* infected mice receiving injections with only vehicle experienced weight loss that began 24 h post infection and continued through 72 h (*Fig 5B*). In the XMU-MP-1 treated group, infected mice experienced weight loss at 24 h and subsequent recovery in weight at 48 and 72 h, indicating XMU-MP-1 attenuated *C*. *difficile* disease. In addition to the experiment presented in *Fig 5B*, two additional independent experiments are presented in *S8 Fig* and each demonstrates XMU-MP-1 ameliorates *C*. *difficile* disease. Further assessment of *C*. *difficile* disease was conducted by histopathological analysis of ceca at 72 h post infection. As shown in representative H&E stained sections, *C*. *difficile* infection revealed submucosal edema and a mixed inflammatory cell infiltrate, while these effects were minimal when infected mice received XMU-MP-1 *(Figs 5C* and *S9*). Additionally, H&E stained sections from 3 separate experiments were unbiasedly scored by a board-certified veterinary pathologist. This pathology scoring system evaluated edema and inflammation and used a 0–4 scale with 4 demonstrating the most severe pathology. As shown in *Fig 5D*, infected mice without XMU-MP-1 displayed edema scores that ranged from 1 to 4 with an average score of 1.7 while XMU-MP-1 treatment reduced the edema score to an average of 0.4. For cecum inflammation, an average score of 2.1 was observed in mice not receiving XMU-MP-1, and this inflammation score was reduced to 0.4 for mice injected with XMU-MP-1 (*Fig 5D*).

We next determined if XMU-MP-1 impacted the colonization of *C*. *difficile* in mice by quantifying *C*. *difficile* CFUs in fecal samples at 24 and 48 h post infection. Results of this analysis verified mice were colonized with *C*. *difficile* in both vehicle control and XMU-MP-1 treated mice (*Fig 5E*) and demonstrated XMU-MP-1 resulted in a reduction in fecal *C*. *difficile* at 48 hours post infection (*Fig 5E*).

## Discussion

As a protein associated with several human cancers and one that is also targeted by a toxin produced by a prominent human pathogen, therapies that reduce CSPG4 expression could be highly beneficial in treating multiple diseases. Indeed, previous work has shown that CSPG4^-/-^ mice are resistant to *C*. *difficile* disease [22], further supporting the notion that reducing levels of this receptor during infection could benefit the host. To this end, a primary goal of this study was to identify key signaling pathways to target and reduce CSPG4 expression.

Since *C*. *difficile* TcdB2 is highly dependent on CSPG4, we reasoned this toxin would be useful for enriching for cells with reduced CSPG4. Moreover, enriching for cells with a dramatic loss in sensitivity to TcdB2, but only marginal changes in susceptibility to TcdB1, provided a strategy to increase the likelihood of selecting for cells with a loss in CSPG4 expression. With this rationale in mind, we implemented an experimental approach that biased the enrichment towards cells with a loss in expression of CSPG4. By confirming the loss of CPSG4 and examining the transcriptional profiles of the TcdB2 resistant cells, we found previously unreported regulators of CSPG4 expression and were successful in targeting one of these pathways to reduce *C*. *difficile* disease severity. To our knowledge, this is an unprecedented method of both identifying regulators of a host-cell receptor and targeting a regulatory pathway to eliminate this receptor during disease.

Generating the HeLa R5 cells required multiple iterations until we found that passaging cells with increasing amounts of TcdB over the course of approximately 4 weeks yielded a TcdB-resistant cell line. Curiously, a small population of surviving cells consistently emerges after treatment with high doses of TcdB, but those cells do not retain their resistance when expanded and further passaged in the absence of the toxin. We suspect the gradient treatment allows the HeLa cells to accumulate multiple genetic changes, which might not be possible in a single higher dose exposure.

HeLa cells frequently undergo chromosomal rearrangements and develop drug resistance [27], and the successful enrichment and selection for TcdB-resistant cells could depend on these characteristics. Karyotype analysis indicated HeLa R5 cells originated from the parental cells, though HeLa R5 cells had undergone notable changes in chromosome copy numbers. The *CSPG4* gene is located on chromosome 15 (cytogenetic band 15q.24.2), which is present as an additional chromosome copy in HeLa R5 cells. In addition to chromosome 15, as summarized in *S4 Fig*, chromosomes 7,9, 10 and X were found to have either gained or lost copies between HeLa and HeLa R5 cells. Thus, while drug resistance in HeLa cells can be a complicating factor, we took advantage of this to find previously undescribed aspects of CSPG4 regulation. Previous screens (insertional mutagenesis, shRNA, CRISPR-Cas9) were designed to find a single target in resistant cells [15,17,18] and are well-suited for determining factors critical for intoxication, such as receptors. The approach used in this study provides a method that can be used in parallel to identify factors regulating receptor expression.

Passaging the HeLa R5 cells for 16 weeks did not cause the cells to regain sensitivity to TcdB2, suggesting a genetically stable mechanism for resistance to the toxin. DNA sequence comparisons between HeLa and HeLa R5 cells did not identify sequence differences in promoter or coding regions of *CSPG4*. In the absence of sequence changes in *CSPG4* and its regulatory regions, we reasoned that the loss of expression was due to alterations in signaling networks that regulate expression of *CSPG4*. Moreover, considering the complexity of chromosomal rearrangements and significant changes in the DNA sequences between HeLa and HeLa R5 cells, the suppression of CSPG4 was unlikely to localize to specific DNA sequence changes. This led us to generate mRNA transcript profiles from HeLa and HeLa R5 cells and examine these for connections to changes in signaling networks. To analyze the transcriptional data in a way that identifies regulatory networks rather than simply a bulk comparison of all transcriptional differences, we took advantage of the IPA tool. This bioinformatics tool uses a curated database to compare transcriptome profiles and identify signatures that correspond to established regulatory networks and upstream effectors of signaling pathways. Based on this data set, we first tested estrogen signaling to confirm the IPA predictions. Hippo signaling was then selected for further analysis and as a potential target for therapeutic intervention.

Hippo signaling has not previously been connected to CPSG4 expression, although the influence of TcdB on Hippo signaling is not entirely unexpected. Accumulation of YAP1/TAZ in the nucleus and association with TEAD transcription factors is impacted by cell structure, mechanical stresses, and levels of polymerized actin [28,29]. Thus, a reasonable prediction is TcdB-mediated inactivation of Rho leads to depletion of F-actin and a decline in active YAP1-mediated transcription. This idea is further supported by a report from Song et al. in which TcdA and TcdB were shown to inactivate YAP1/TAZ in two cell types [30]. The underlying connections between acquired resistance through loss of CSPG4 expression and alterations in YAP1/TAZ activity is the subject on ongoing studies in our laboratory. Indeed, the data suggest an additional step in CSPG4 regulation remains to be identified. That is, a YAP1/TAZ-regulated gene product may repress CSPG4 or YAP1/TAZ may form a repressor complex that directly inhibits CSPG4 expression. Finally, we also note that the inhibitory effects of XMU-MP-1 were found to be stronger on HT-29 cells compared to HeLa cells. Many factors could contribute to the effectiveness of XMU-MP-1 in HT-29 cells, but one important observation is that HT-29 cells lack *ESR1* expression [31]. Unlike HeLa cells, HT-29 cells may primarily rely on Hippo signaling to regulate CSPG4. The ability to fully repress CSPG4 expression by targeting a single regulatory pathway will likely depend on whether the gene is regulated by one or more systems in a particular cell type.

We predicted that suppressing CSPG4 expression in vivo by targeting the Hippo pathway could protect *C*. *difficile* infected mice from TcdB2. The MST1/2 inhibitor XMU-MP-1 was selected because this small molecule has an exceptional pharmacokinetics profile and was used to modulate Hippo signaling in various experimental mouse models [24]. Treating infected mice with XMU-MP-1 both reduced CSPG4 levels in the colon and attenuated *C*. *difficile* disease (Fig 5). The dose and administration schedule of XMU-MP-1 was based the work by Fan et al. [24], and we suspect that further fine tuning XMU-MP-1 administration could further improve outcomes in *C*. *difficile* disease. Overall, this finding supports the concept of targeting expression of a receptor in order to prevent toxin-mediated disease effects. Our work not only has identified a critical pathway for regulating CSPG4 but may have also discovered a new approach for combating infectious disease and possibly reducing the use of antibiotics.

## Materials and methods

### Institutional compliance

All experiments were carried out in accordance with relevant guidelines and regulations. All animal procedures were carried out with approval from the University of Oklahoma Health Sciences Center Institutional Animal Care and Use Committee under protocol number 18-049-HI. The procedures used in this study strictly adhered to the guidelines found in the National Research Council’s *Guide for the Care and Use of Laboratory Animals*.

### Cell culture and reagents

Cells lines used in this study were purchased from American Type Culture Collection. HeLa-CCL2 cells were cultured in Eagle’s Minimum Essential Medium supplemented with 10% FBS, 100 units/ml penicillin, and 100 μg/ml streptomycin. The human colorectal cell line HT-29 was cultured in McCoy’s 5a media supplemented with 10% FBS, 100 units/ml penicillin, and 100 μg/ml streptomycin. All cells were grown at 37°C in the presence of 5% CO_2_.

The following reagents were purchased from Sigma: Encapsulated β-Estradiol (product # E4389), β-Estradiol (product # E8875), and XMU-MP-1 (product # SML2233). TRULI (product # HY-138489) was purchased from MedChemExpress.

### Recombinant toxins

TcdB1 and TcdB2 were expressed and purified in a *Bacillus megaterium* recombinant system as previously described [32]. TcdB1^FZD-^ was created in pC-His1622-TcdB1 using the NEBuilder HiFi DNA Assembly Master Mix (New England Biolabs), then expressed and purified using the *B*. *megaterium* system. Recombinant TcdA was obtained as a gift from Dr. Borden Lacy.

### Generation of TcdB2 resistant HeLa cells (HeLa R5)

HeLa cells were seeded into 12-well plates at a density of 2.5 × 10^5^ cells per well and cultured overnight. These cells were then exposed to 0.02 pM of TcdB2, and levels of TcdB2 were increased in step-wise increments over the course of a month until reaching a final concentration of 20 pM. Approximately every three days (depending on the extent of visualized cell death), the cultures were washed with media to remove dead and detached cells, and then fresh media containing TcdB2 was added to the cells. After >90% of non-rounding cells were obtained at the 20 pM concentration, TcdB2 resistant cells (HeLa R5) were subcultured into T-25 tissue culture flasks. HeLa R5 cells were routinely maintained under selective pressure with TcdB2 added to the culture medium. Resistance stability in the absence of TcdB2 was determined by passage of the HeLa R5 cells for 16 weeks without the toxin. The cells were then assayed for TcdB2-sensitivity and compared to baseline HeLa cells sensitivity to the toxin.

### Karyotype analysis

Cytogenetic analyses for HeLa cells were performed using standardized protocols of the Genetics Lab at The University of Oklahoma Health Sciences Center. Twenty metaphase cells were examined for the HeLa cell line and the HeLa R5 cell line, and karyotype was described according to the International System for Human Cytogenetic Nomenclature 2016 [33]. Also, previously reported cytogenetic characterization of the HeLa cells [34] was used as a reference to determine complex chromosomal abnormalities.

### Quantification of cytopathic effects (CPE)

Cells were seeded in 96-well tissue culture plates at a density of 1x10^4^ cells per well. The following day, cells were exposed for 24 h to a range of toxin concentrations and then the cells were fixed for 10 min with phosphate-buffered saline (PBS) containing 4% formaldehyde. After imaging cells from several fields, both nonrounded and rounded cells were enumerated and % CPE was calculated as [(# rounded cells/total cell #)*100].

### Microscale thermophoresis

Microscale thermophoresis (MST) experiments to confirm the absence of FZD binding were performed using NanoTemper Monolith NT.115 (NanoTemper Technologies Gmbh, Munich, Germany). TcdB1, TcdB1^FZD-^, and TcdB2 were labeled with the Monolith NT His-tag Labeling Kit RED-Tris-NTA (Cat. #MO-L018), following the manufacturer’s instructions. A fixed concentration of 50 nM TcdB1, TcdB1^FZD-^, or TcdB2, respectively, was titrated with 315 μM to 0.82 μM of FZD 7 (R&D Systems) in PBS (pH 7.4). Samples were loaded into Monolith NT.115 premium capillaries (NanoTemper) and measurements were carried out at 37°C with 40% MST power and 60% excitation power. Data were collected with MO.Control v1.5.3, analyzed with MO.Affinity Analysis v2.2.7, and graphed in Prism v9.2.

### Flow cytometry and immunoblot analysis

For analysis of CSPG4 levels, suspended cells were incubated for 1 h on ice with 20 μg/ml of a commercially available mixture of four monoclonal antibodies to CPSG4 (Thermo Fisher Scientific, product #37–2700). Primary antibody was detected using an anti-mouse secondary antibody labeled with Alexa Fluor 647 (Cell Signaling Tech, product # 4410). For TcdB binding experiments, TcdB1 and TcdB2 were each labeled on primary amines with Alexa Fluor 488 (ThermoFisher, product #A10235). Alexa Fluor 488-labeled TcdB1(TcdB1_AF488_) or TcdB2 (TcdB2_AF488_) was then added to suspended cells and binding after 30 min on ice was measured. A FACSCalibur flow cytometer (Laboratory of Molecular Biology and Cytometry Research, University of Oklahoma Health Sciences Center) was used to measure CSPG4 antibody binding and TcdB binding to cells. Flow cytometry data was analyzed using FLOWJO software (Tree Star).

Immunoblot analysis of cell culture lysates was performed as previously described [35]. Primary antibodies used for immunoblots in this study were a mouse monoclonal antibody recognizing nonglucosylated Rac1 (BD Bioscience; catalog no. 610651); a mouse monoclonal antibody recognizing total Rac1 (EMD Millipore; catalog no. 05–389); a mouse monoclonal antibody against GAPDH (Abcam; product # ab8245); a mouse monoclonal against CSPG4 (R&D Biosystems; product # MAB2585); a mouse monoclonal against YAP1 (Santa Cruz Biotechnology; product # sc-101199); and a rabbit monoclonal against TFPI (Abcam; product # ab260041). A rabbit monoclonal antibody (Cell Signaling Technology; product # 4970) was used in a ProteinSimple capillary separation immunodetection system (Bio-Techne) to detect ß-actin from lysates in the HeLa and HeLa YAP1 deletion mutant.

### RT-qPCR

From HeLa, HeLa R5, or HT-29 cells, RNA was isolated then converted to cDNA in reverse transcription reactions using SuperScript III (Invitrogen). The resulting cDNA was combined with a SYBR Green PCR master mix (Qiagen) as well as gene-specific primers. Amplification reactions were then performed with an Applied Biosystems QuantStudio 5 real-time PCR system. Relative changes in levels of the mRNA of the gene of interest were compared with the levels of human *ACTB* mRNA using the 2^−ΔΔCt^ method.

### Transfection of HeLa R5 cells

To express CSPG4, HeLa R5 cells were transfected with the plasmid pEF6-CSPG4-myc-his (Addgene plasmid # 69037; http://n2t.net/addgene:69037, Wengheng Wei) [15]. To transfect this plasmid into HeLa R5 cells, the cells were plated at 1x10^5^ cells per well of a 12 well tissue culture plate in EMEM with 10% FBS and allowed to grow overnight. The following day, a transfection mixture containing 4 μL of FuGENE HD (Promega) and 1 μg of plasmid DNA (4,1 ratio of FuGENE HD to DNA) was prepared in 50 μL tissue-culture media (no FBS or antibiotic). This was then added to one well of a 12 well tissue culture plate containing the HeLa R5 cells cultured in EMEM with 10% FBS. After a 5 h exposure, the media was removed and replaced with fresh media. TcdB2 treatments were carried out between 24 h and 48 h after transfection.

### Generation of YAP1 knockout

Using the protocol described for the plasmid pEF6-CSPG4-myc-his, HeLa cells were transfected with a mixture of two YAP1 double nickase plasmids (Santa Cruz Biotechnology; sc-400040-NIC) with each containing a different gRNA sequence. Single cell clones were isolated and screened for YAP1 deletion by immunoblot.

### Whole genome sequencing

HeLa and HeLa R5 cells were pelleted by centrifugation and then frozen in liquid N_2_. Whole genome sequencing (WGS) was performed by Omega Services (Norcross, GA) with library preparation using a KAPA HyperPlus kit (Roche) and sequencing performed with Illumina NovaSeq 6000.

### RNA-Seq and Ingenuity Pathway Analysis

RNA was isolated from HeLa and HeLa R5 cells using Direct-Zol microprep (Zymogen) and suspended in RNase-free water. Overall RNA purity and concentration was measured using an Agilent Bioanalyzer (Agilent Technology). Total RNA (200 ng—500 ng) was provided to the Genomics Core at the University of Oklahoma Health Sciences Center Institutional Research Core Facility and used to prepare mRNA template libraries that were sequenced using 2 x 150 bp reads on an Illumina NovaSeq 6000. Derived sequences were mapped to the human genome (GRCh38) by gSNAP [36], expression (FPKM) derived by Cufflinks [37], and differential expression determined as a +/-1.5-fold change from control. Genes with significant fold changes from control were submitted to pathway analysis using Ingenuity Pathway Analysis (Qiagen) to identify pathways of interest.

### *C*. *difficile* infection in a mouse model

The impact of XMU-MP-1 on *C*. *difficile* infections was tested in the cefoperazone mouse model of *C*. *difficile* infection [26]. In this model, eight-week-old C57BL/6J female mice were given sterile drinking water containing 0.5 mg/ml of cefoperazone for 5 days, followed by 2 days of sterile drinking water without cefoperazone. After this antibiotic exposure protocol, *C*. *difficile* (R20291) spores were given to mice by oral gavage of 10^6^ spores in 50 μl of sterile water. To administer XMU-MP-1, mice underwent daily intraperitoneal injections of 1 mg/kg of XMU-MP-1 that began 2 days before *C*. *difficile* infection and continued through the course of the experiment. XMU-MP-1 was prepared by dissolving in DMSO at a concentration of 25 mg/ml and then diluting this stock to 100 μg/ml in the injection vehicle solution consisting of PBS with 20% Kolliphor HS 15 and 0.1% citric acid. Through the course of the experiment, the health of the mice was monitored, weight loss was tracked, and fecal pellets were collected. At 96 h post-challenge, mice were humanely euthanized by CO_2_ inhalation and the ceca and colons were removed for histopathological evaluation and immunofluorescence staining.

### Histopathology

Ceca were excised from mice and immediately placed in 10% neutral buffered formalin. The fixed tissue was paraffin embedded, sectioned, and then hematoxylin & eosin (H&E) stained at the OUHSC Cell Biology Histology Core. Images of the H&E stained sections were captured using a brightfield microscope (Olympus B40x) equipped with a SPOT 5 MP digital camera with SPOT 5.3 imaging software (Sterling Heights, MI). An unbiased evaluation of the histopathology of each treatment (n = 14–16) was carried out by a board-certified veterinary pathologist that scored edema and inflammation in a manner similar to previous studies [38].

### Immunofluorescence

Colons were removed from mice, Swiss-rolled, and submerged in 4% paraformaldehyde overnight at 4°C. The following day, colons were washed five times and placed in a 15% sucrose-PBS solution overnight, then moved into a 30% sucrose-PBS solution for 24 h. The Swiss-rolled colons were embedded in optimal cutting temperature compound (OCT) and sectioned (20-μm thickness). The colon sections were blocked (PBS containing 10% goat serum, 5% BSA, and 0.5% triton X-100) and incubated overnight at room temperature with an Alexa Fluor 488 labeled rat monoclonal antibody against E-cadherin (Santa Cruz Biotechnology; sc-59778 AF488) and rabbit antibodies against CSPG4 (Abcam; ab83178). After 3 washes, the sections were incubated with a Cy3 labeled goat anti-rabbit antibody (Abcam; ab6939) for 1 h at room temperature, washed, and mounted in Prolong Gold with DAPI. Stained sections were imaged on a Nikon W1-CSU/SoRa super-resolution confocal microscope using a 10X objective. For each channel, an equivalent amount of background was subtracted using Nikon NIS Elements v4.2. Brightness and contrast were applied equally for all images using ImageJ v2.1.0.

### Colony forming units in mouse feces

Fecal samples were collected from mice at day 0 and days 1 and 2 postgavage. Samples were diluted 1:10 (wt/vol) in PBS and homogenized. Serial dilutions [1,10] were made anaerobically and one hundred microliters of each dilution was plated on taurocholate-cycloserine-cefoxitin-fructose agar. The plates were incubated at 37°C for 24 h before the colonies were enumerated. Calculation of the total number of colony forming units (CFU) was based on the dilution factor and the initial weight of the feces collected. The dilution plate with the lowest countable number (between 20 and 200 colonies) was used for calculation of the number of CFU per gram of fecal content.

## Supporting information

S1 Fig Microscale thermophoresis confirms decreased FZD binding for TcdB1 ^FZD-^. MST binding curves of TcdB1 (blue), TcdB1 ^FZD-^, and TcdB2 to FZD 7. Data was normalized and the dissociation constant was calculated using the K_D_ slope model (law of mass action). ΔFnorm[‰] represents the relative change in normalized fluorescence per thousand. K_D_ for TcdB1 at 37°C was 7.71 ± 7.57 nM. K_D_ for TcdB1 ^FZD-^ and TcdB2 were not detected. Data are represented as mean ± SEM (n = 2).
(PDF)

S2 Fig TFPI levels in HeLa and HeLa R5 cells. Immunoblot analysis of TFPI and GAPDH levels using protein lysates from HeLa and HeLa R5 cells.
(PDF)

S3 Fig CSPG4 levels in HeLa R5 cells passaged for 4 or 16 weeks in the absence of TcdB2. CSPG4 transcript levels were determined by RT-qPCR and are presented as the mean (n  =  3) ± S.D.
(PDF)

S4 Fig Karyogram analysis of HeLa and HeLa R5 cells. Both HeLa cells revealed a composite karyotype including 74~83 chromosomes (hypertriploidy) with recurrent structural and numerical chromosomal anomalies. Diagonal black arrows show structural abnormalities detected. Both HeLa cell lines showed almost similar structural abnormalities. Green and Red arrows indicate changes in numbers of individual chromosomes between HeLa and HeLa R5 cells. The HeLa R5 cells had different percentages of copy number changes, copy number gain of chromosomes 7 and 15, and copy number loss of chromosomes X, 9, and 10 compared to parental HeLa cells. The chromosomal analysis of the HeLa (before treatment) and the HeLa R5 (after treatment) revealed a composite karyotype, including 74~83 chromosomes (hypertriploidy) with structural and numerical anomalies. Both cell lines had almost the same structural abnormalities as follows: der [1]t(1;3)(q11;q11),der(1;9)(p10;q10),dup [2](q?q?),der(3;5)(p10;q10),der(3;20)(q10;q?10),der [5]t(5;22;8)(q11;q11q13;?),i [5](p10),der [7]t(7;19)(q35;?),del [7](p21),der [9]t(3;9)(p21;p11),der(5;9)(p10;p10),i [9](p10),der [11]t(9;11;9)(?;p14?q22?;?)dup [11](p?)dup [11](q?),der [12]t(3;12)(q21;q15),i [15](q10),der [16]t(7;16)(p21?;p11), der [19]t(13;19)(q21;p13), i [20](q10), der [22]t(8;22)(?;q13). In addition to the structural abnormalities, chromosomes X, 7, 9, 10, 15 showed percentage differences in the copy numbers. For chromosomes 7 and 15, trisomy percentages were increased from 0% and 65% in the HeLa to 75% and 95% in the HeLa R5, respectively. For chromosomes X, 9, and 10, trisomy percentages were decreased from 70%, 85%, and 100% in the HeLa to 10%, 5%, and 25% in the HeLa R5, respectively.
(PDF)

S5 Fig TcdB2 activity on HeLa R5 cells exposed to a DNA methylation inhibitor. Cytotoxicity assay quantifying cytopathic effects (CPE) after 24 h exposure to TcdB2 in HeLa R5 cells with and without a 48 h exposure to 5 μM of 5-AZA-CdR. Data are presented as mean (n  =  3) ± S.D. *p < 0.05 determined by Student’s t-test.
(PDF)

S6 Fig Densitometry analysis of CSPG4 immunoblots from Figs 3 and 4. (*A*) HeLa cells were exposed to 10 μg/ml of encapsulated ß-estradiol for 24 h. An immunoblot was performed and CSPG4 was quantified from multiple immunoblot lanes. The relative band density is presented as mean (n  =  4) ± S.D. (*B-C*) Hela cells were treated with 6 μM XMU-MP-1 or 30 μM of TRULI for 24 h. Immunoblots were carried out and CSPG4 was quantified from immunoblots. Relative band density is presented as mean (n  =  3) ± S.D. (*D-E*) HT-29 cells were treated with 6 μM XMU-MP-1 or 30 μM of TRULI for 24. Immunoblots were performed and CSPG4 was quantified from immunoblots. Relative band density is presented as mean (n  =  2) ± S.D. *p < 0.05 determined by Student’s t-test.
(PDF)

S7 Fig Mouse model of *C*. *difficile* infection and administration schedule for XMU-MP-1. (PDF)

S8 Fig *C*. *difficile* disease in XMU-MP-1 treated mice from independent experiments. Mice were infected with *C*. *difficile* strain R20291 and received daily intraperitoneal injections with 1 mg/kg of XMU-MP-1 or vehicle starting 2 days before infection and continuing through the length of the experiment. (*A*) Percent change in weight in mice post infection presented as mean (n  =  5) ± S.D. (*B*) Percent change in weight in mice post infection presented as mean (n  =  6) ± S.D. *p < 0.05 indicates significance between vehicle and XMU-MP-1 treatment.
(PDF)

S9 Fig Histology of ceca from C. difficile infected mice. Mice were infected with *C*. *difficile* strain R20291 and received daily intraperitoneal injections with 1 mg/kg of XMU-MP-1 or vehicle starting 2 days before infection and continuing through the length of the experiment. Mice were euthanized at 96 h post infection and excised ceca were H&E stained.
(PDF)

S1 Table *In vitro* growth of *C*. *difficile* strain R20291 in the presence and absence of XMU-MP-1. (PDF)
