# Supplementary material for: Discovery of Hippo signaling as a regulator of CSPG4 expression and as a therapeutic target for Clostridioides difficile disease
Source: PLoS Pathog. 2023 Mar 27;19(3):e1011272. doi: 10.1371/journal.ppat.1011272 (PMC10079225; doi:10.1371/journal.ppat.1011272)
Supplement: S1 Fig — MST binding curves of TcdB1 (blue), TcdB1 FZD-, and TcdB2 to FZD 7. Data was normalized and the dissociation constant was calculated using the KD slope model (law of mass action). ΔFnorm[‰] represents the relative change in normalized fluorescence per thousand. KD for TcdB1 at 37°C was 7.71 ± 7.57 nM. KD for TcdB1 FZD- and TcdB2 were not detected. Data are represented as mean ± SEM (n = 2). (PDF) [file ppat.1011272.s001.pdf]

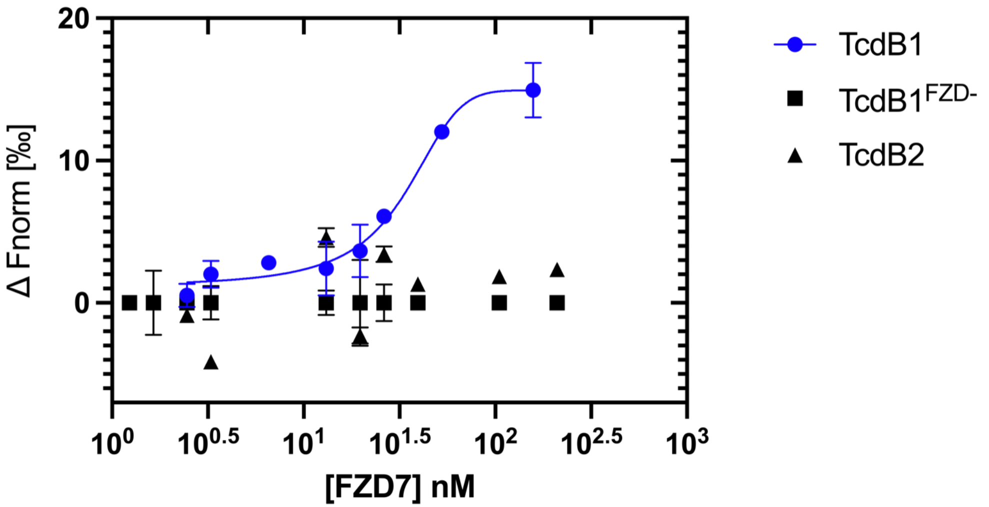

S1 Fig. Microscale thermophoresis confirms decreased frizzed binding for TcdB1 $^{FZD-}$. MST binding curves of TcdB1 (blue), TcdB1 $^{FZD-}$, and TcdB2 to FZD 7. Data was normalized and the dissociation constant was calculated using the K_D slope model (law of mass action). ΔFnorm[‰] represents the relative change in normalized fluorescence per thousand. K_D for TcdB1 at 37°C was 7.71 ± 7.57 nM. K_D for TcdB1 $^{FZD-}$ and TcdB2 were not detected. Data are represented as mean ± SEM (n=2).
