# Supplementary material for: Discovery of Hippo signaling as a regulator of CSPG4 expression and as a therapeutic target for Clostridioides difficile disease
Source: PLoS Pathog. 2023 Mar 27;19(3):e1011272. doi: 10.1371/journal.ppat.1011272 (PMC10079225; doi:10.1371/journal.ppat.1011272)
Supplement: S2 Fig — Immunoblot analysis of TFPI and GAPDH levels using protein lysates from HeLa and HeLa R5 cells. (PDF) [file ppat.1011272.s002.pdf]

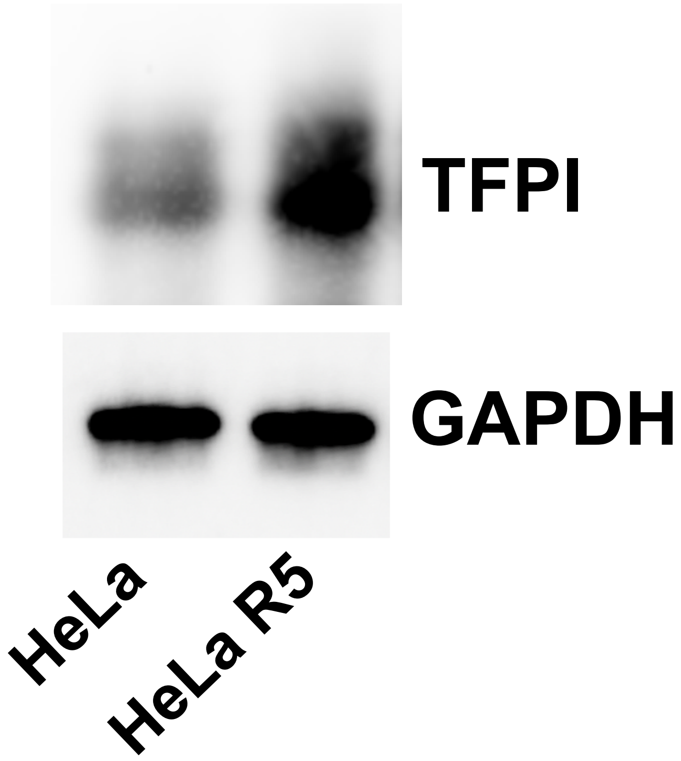

S2 Fig. TFPI levels in HeLa and HeLa R5 cells. Immunoblot analysis of TFPI and GAPDH levels using protein lysates from HeLa and HeLa R5 cells.
