# Supplementary material for: Discovery of Hippo signaling as a regulator of CSPG4 expression and as a therapeutic target for Clostridioides difficile disease
Source: PLoS Pathog. 2023 Mar 27;19(3):e1011272. doi: 10.1371/journal.ppat.1011272 (PMC10079225; doi:10.1371/journal.ppat.1011272)
Supplement: S3 Fig — CSPG4 transcript levels were determined by RT-qPCR and are presented as the mean (n = 3) ± S.D. (PDF) [file ppat.1011272.s003.pdf]

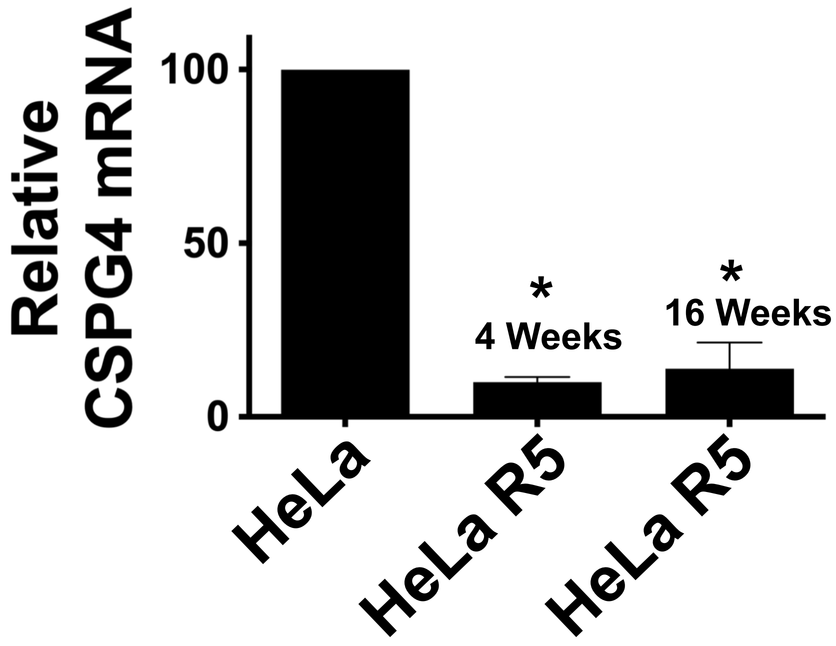

S3 Fig. CSPG4 levels in HeLa R5 cells passaged for 4 or 16 weeks in the absence of TcdB2. CSPG4 transcript levels were determined by RT-qPCR and are presented as the mean (n = 3) ± S.D.
