# Supplementary material for: Discovery of Hippo signaling as a regulator of CSPG4 expression and as a therapeutic target for Clostridioides difficile disease
Source: PLoS Pathog. 2023 Mar 27;19(3):e1011272. doi: 10.1371/journal.ppat.1011272 (PMC10079225; doi:10.1371/journal.ppat.1011272)
Supplement: S4 Fig — Both HeLa cells revealed a composite karyotype including 74~83 chromosomes (hypertriploidy) with recurrent structural and numerical chromosomal anomalies. Diagonal black arrows show structural abnormalities detected. Both HeLa cell lines showed almost similar structural abnormalities. Green and Red arrows indicate changes in numbers of individual chromosomes between HeLa and HeLa R5 cells. The HeLa R5 cells had different percentages of copy number changes, copy number gain of chromosomes 7 and 15, and copy number loss of chromosomes X, 9, and 10 compared to parental HeLa cells. The chromosomal analysis of the HeLa (before treatment) and the HeLa R5 (after treatment) revealed a composite karyotype, including 74~83 chromosomes (hypertriploidy) with structural and numerical anomalies. Both cell lines had almost the same structural abnormalities as follows: der [1]t(1;3)(q11;q11),der(1;9)(p10;q10),dup [2](q?q?),der(3;5)(p10;q10),der(3;20)(q10;q?10),der [5]t(5;22;8)(q11;q11q13;?),i [5](p10),der [7]t(7;19)(q35;?),del [7](p21),der [9]t(3;9)(p21;p11),der(5;9)(p10;p10),i [9](p10),der [11]t(9;11;9)(?;p14?q22?;?)dup [11](p?)dup [11](q?),der [12]t(3;12)(q21;q15),i [15](q10),der [16]t(7;16)(p21?;p11), der [19]t(13;19)(q21;p13), i [20](q10), der [22]t(8;22)(?;q13). In addition to the structural abnormalities, chromosomes X, 7, 9, 10, 15 showed percentage differences in the copy numbers. For chromosomes 7 and 15, trisomy percentages were increased from 0% and 65% in the HeLa to 75% and 95% in the HeLa R5, respectively. For chromosomes X, 9, and 10, trisomy percentages were decreased from 70%, 85%, and 100% in the HeLa to 10%, 5%, and 25% in the HeLa R5, respectively. (PDF) [file ppat.1011272.s004.pdf]

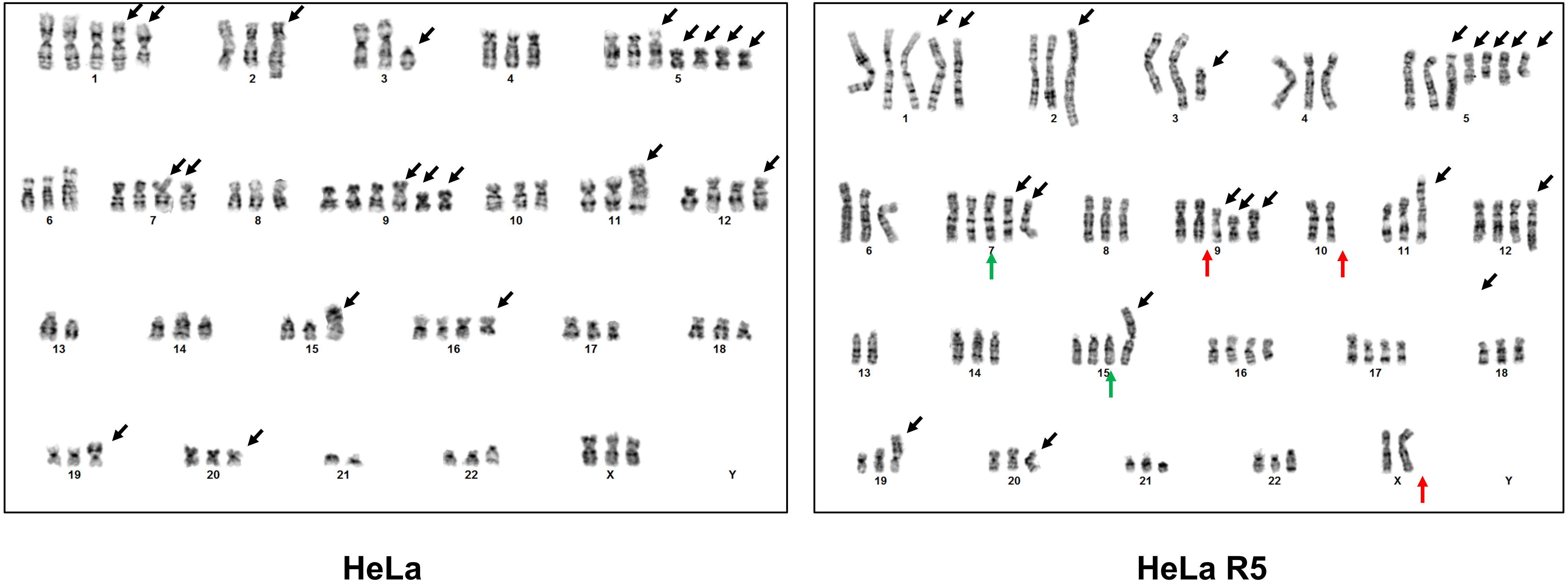

S4 Fig. Karyogram analysis of HeLa and HeLa R5 cells. Both HeLa cells revealed a composite karyotype including 74~83 chromosomes (hypertriploidy) with recurrent structural and numerical chromosomal anomalies. Diagonal black arrows show structural abnormalities detected. Both HeLa cell lines showed almost similar structural abnormalities. Green and Red arrows indicate changes in numbers of individual chromosomes between HeLa and HeLa R5 cells. The HeLa R5 cells had different percentages of copy number changes, copy number gain of chromosomes 7 and 15, and copy number loss of chromosomes X, 9, and 10 compared to parental HeLa cells. The chromosomal analysis of the HeLa (before treatment) and the HeLa R5 (after treatment) revealed a composite karyotype, including 74~83 chromosomes (hypertriploidy) with structural and numerical anomalies. Both cell lines had almost the same structural abnormalities as follows: der(1)t(1;3)(q11;q11),der(1;9)(p10;q10),dup(2)(q?q?),der(3;5)(p10;q10),der(3;20)(q10;q?10),der(5)t(5;22;8)(q11;q11q13;?),i(5)(p10),der(7)t(7;19)(q35;?),del(7)(p21),der(9)t(3;9)(p21;p11),der(5;9)(p10;p10),i(9)(p10),der(11)t(9;11;9)(?;p14?q22?;?)dup(11)(p?)dup(11)(q?),der(12)t(3;12)(q21;q15),i(15)(q10),der(16)t(7;16)(p21?;p11), der(19)t(13;19)(q21;p13), i(20)(q10), der(22)t(8;22)(?;q13). In addition to the structural abnormalities, chromosomes X, 7, 9, 10, 15 showed percentage differences in the copy numbers. For chromosomes 7 and 15, trisomy percentages were increased from 0% and 65% in the HeLa to 75% and 95% in the HeLa R5, respectively. For chromosomes X, 9, and 10, trisomy percentages were decreased from 70%, 85%, and 100% in the HeLa to 10%, 5%, and 25% in the HeLa R5, respectively.
