# Supplementary material for: Discovery of Hippo signaling as a regulator of CSPG4 expression and as a therapeutic target for Clostridioides difficile disease
Source: PLoS Pathog. 2023 Mar 27;19(3):e1011272. doi: 10.1371/journal.ppat.1011272 (PMC10079225; doi:10.1371/journal.ppat.1011272)
Supplement: S5 Fig — Cytotoxicity assay quantifying cytopathic effects (CPE) after 24 h exposure to TcdB2 in HeLa R5 cells with and without a 48 h exposure to 5 μM of 5-AZA-CdR. Data are presented as mean (n = 3) ± S.D. *p < 0.05 determined by Student’s t-test. (PDF) [file ppat.1011272.s005.pdf]

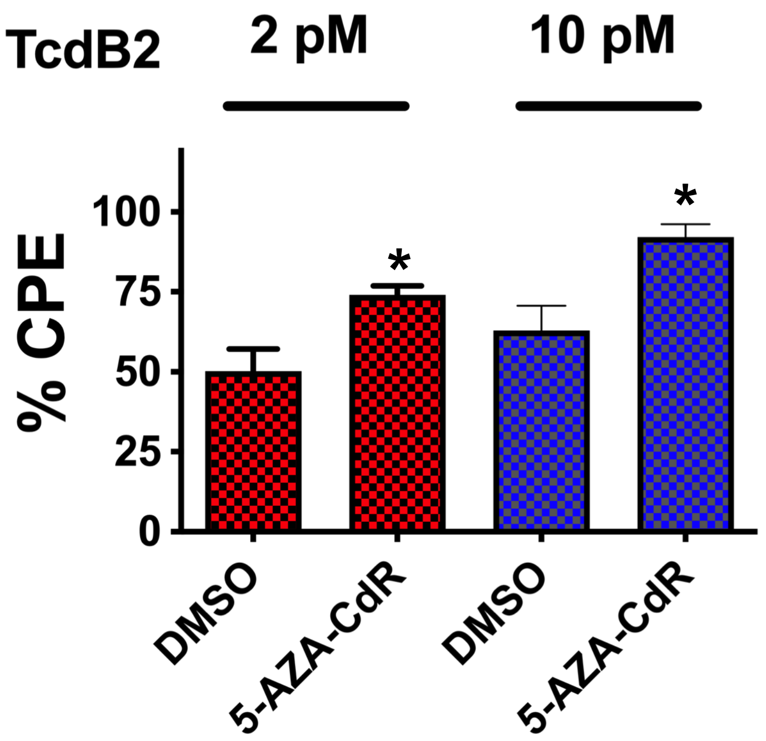

S5 Fig. TcdB2 activity on HeLa R5 cells exposed to a DNA methylation inhibitor. Cytotoxicity assay quantifying cytopathic effects (CPE) after 24 h exposure to TccB2 in HeLa R5 cells with and without a 48 h exposure to 5 μM of 5-AZA-CdR. Data are presented as mean (n = 3) ± S.D. *$p < 0.05$ determined by Student's t-test.
