# Supplementary material for: Discovery of Hippo signaling as a regulator of CSPG4 expression and as a therapeutic target for Clostridioides difficile disease
Source: PLoS Pathog. 2023 Mar 27;19(3):e1011272. doi: 10.1371/journal.ppat.1011272 (PMC10079225; doi:10.1371/journal.ppat.1011272)
Supplement: S6 Fig — (A) HeLa cells were exposed to 10 μg/ml of encapsulated ß-estradiol for 24 h. An immunoblot was performed and CSPG4 was quantified from multiple immunoblot lanes. The relative band density is presented as mean (n = 4) ± S.D. (B-C) Hela cells were treated with 6 μM XMU-MP-1 or 30 μM of TRULI for 24 h. Immunoblots were carried out and CSPG4 was quantified from immunoblots. Relative band density is presented as mean (n = 3) ± S.D. (D-E) HT-29 cells were treated with 6 μM XMU-MP-1 or 30 μM of TRULI for 24. Immunoblots were performed and CSPG4 was quantified from immunoblots. Relative band density is presented as mean (n = 2) ± S.D. *p < 0.05 determined by Student’s t-test. (PDF) [file ppat.1011272.s006.pdf]

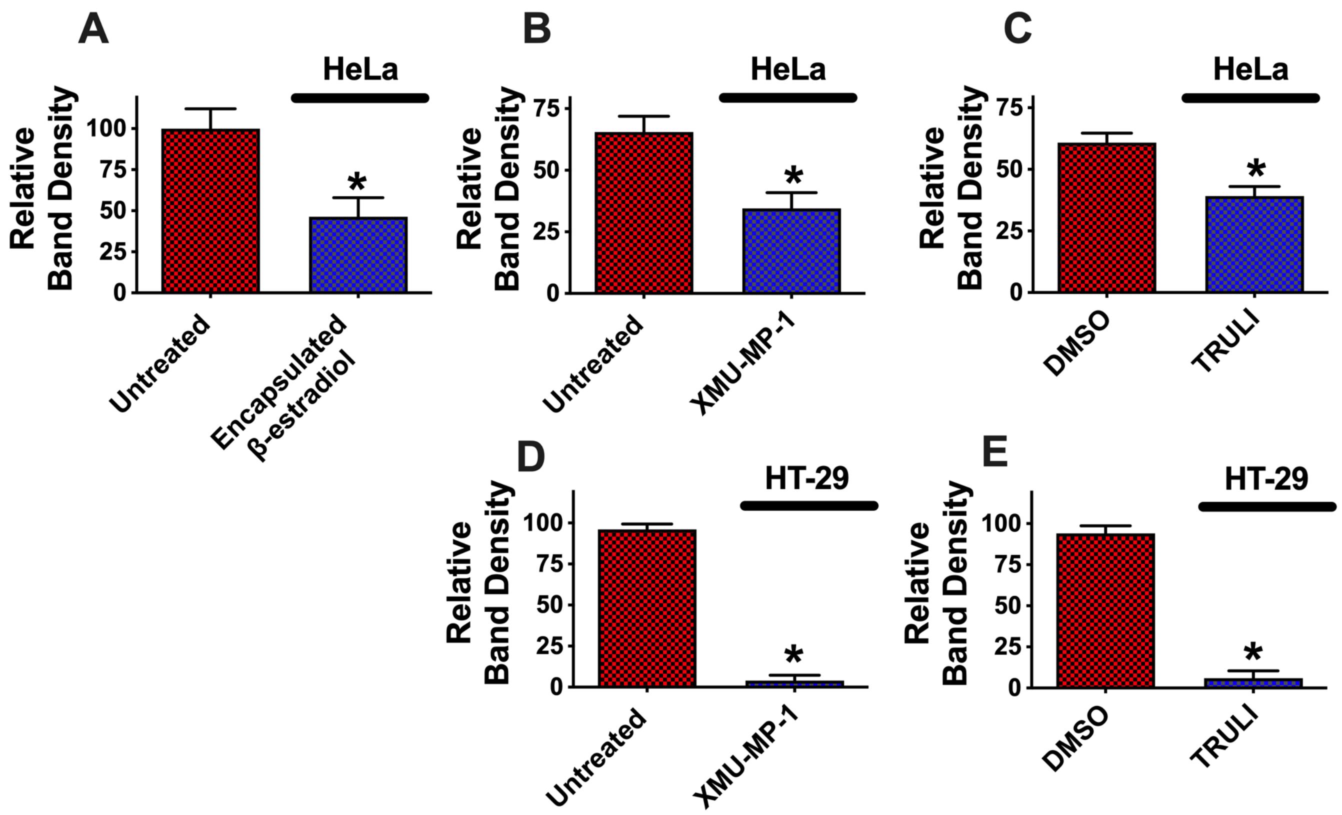

S6 Fig. Densitometry analysis of CSPG4 immunoblots from Fig. 3 and Fig. 4. (*A*) HeLa cells were exposed to 10 μg/ml of encapsulated ß-estradiol for 24 h. An immunoblot was performed and CSPG4 was quantified from multiple immunoblot lanes. The relative band density is presented as mean (n = 4) ± S.D. (*B-C*) Hela cells were treated with 6 μM XMU-MP-1 or 30 μM of TRULI for 24 h. Immunoblots were carried out and CSPG4 was quantified from immunoblots. Relative band density is presented as mean (n = 3) ± S.D. (*D-E*) HT-29 cells were treated with 6 μM XMU-MP-1 or 30 μM of TRULI for 24. Immunoblots were performed and CSPG4 was quantified from immunoblots. Relative band density is presented as mean (n = 2) ± S.D. *$p < 0.05$ determined by Student's t-test.
