# Supplementary figures and images for: Discovery of Hippo signaling as a regulator of CSPG4 expression and as a therapeutic target for Clostridioides difficile disease
Source: PLoS Pathog. 2023 Mar 27;19(3):e1011272. doi: 10.1371/journal.ppat.1011272 (PMC10079225; doi:10.1371/journal.ppat.1011272)

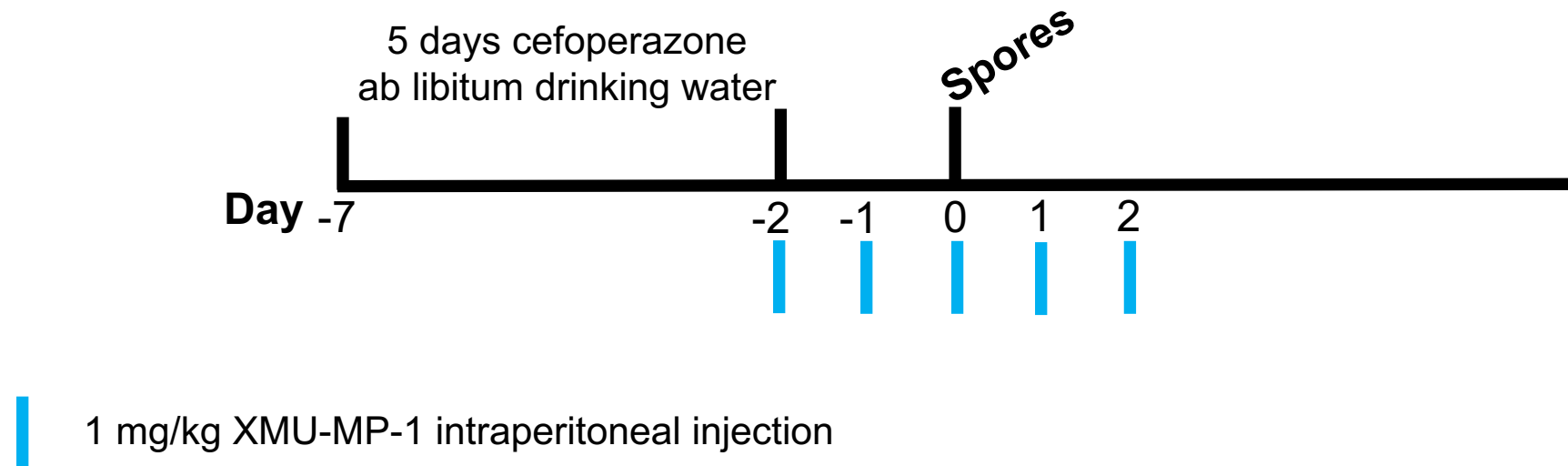

S7 Fig. Mouse model of *C. difficile* infection and administration schedule for XMU-MP-1.

Supplement: S7 Fig — (PDF) [file ppat.1011272.s007.pdf]
