# Supplementary material for: Discovery of Hippo signaling as a regulator of CSPG4 expression and as a therapeutic target for Clostridioides difficile disease
Source: PLoS Pathog. 2023 Mar 27;19(3):e1011272. doi: 10.1371/journal.ppat.1011272 (PMC10079225; doi:10.1371/journal.ppat.1011272)
Supplement: S8 Fig — Mice were infected with C. difficile strain R20291 and received daily intraperitoneal injections with 1 mg/kg of XMU-MP-1 or vehicle starting 2 days before infection and continuing through the length of the experiment. (A) Percent change in weight in mice post infection presented as mean (n = 5) ± S.D. (B) Percent change in weight in mice post infection presented as mean (n = 6) ± S.D. *p < 0.05 indicates significance between vehicle and XMU-MP-1 treatment. (PDF) [file ppat.1011272.s008.pdf]

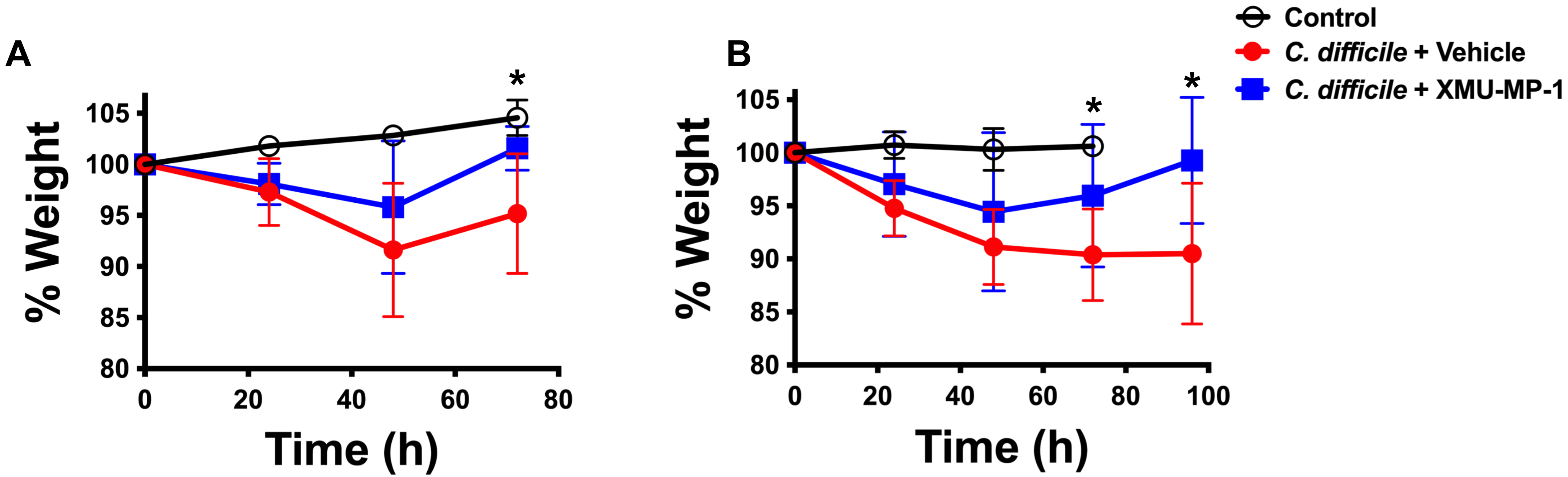

S8 Fig. *C. difficile* disease in XMU-MP-1 treated mice from independent experiments. Mice were infected with *C. difficile* strain R20291 and received daily intraperitoneal injections with 1 mg/kg of XMU-MP-1 or vehicle starting 2 days before infection and continuing through the length of the experiment. (*A*) Percent change in weight in mice post infection presented as mean (n = 5) ± S.D. (*B*) Percent change in weight in mice post infection presented as mean (n = 6) ± S.D. *p < 0.05 indicates significance between vehicle and XMU-MP-1 treatment.
