# Supplementary material for: Discovery of Hippo signaling as a regulator of CSPG4 expression and as a therapeutic target for Clostridioides difficile disease
Source: PLoS Pathog. 2023 Mar 27;19(3):e1011272. doi: 10.1371/journal.ppat.1011272 (PMC10079225; doi:10.1371/journal.ppat.1011272)
Supplement: S9 Fig — Mice were infected with C. difficile strain R20291 and received daily intraperitoneal injections with 1 mg/kg of XMU-MP-1 or vehicle starting 2 days before infection and continuing through the length of the experiment. Mice were euthanized at 96 h post infection and excised ceca were H&E stained. (PDF) [file ppat.1011272.s009.pdf]

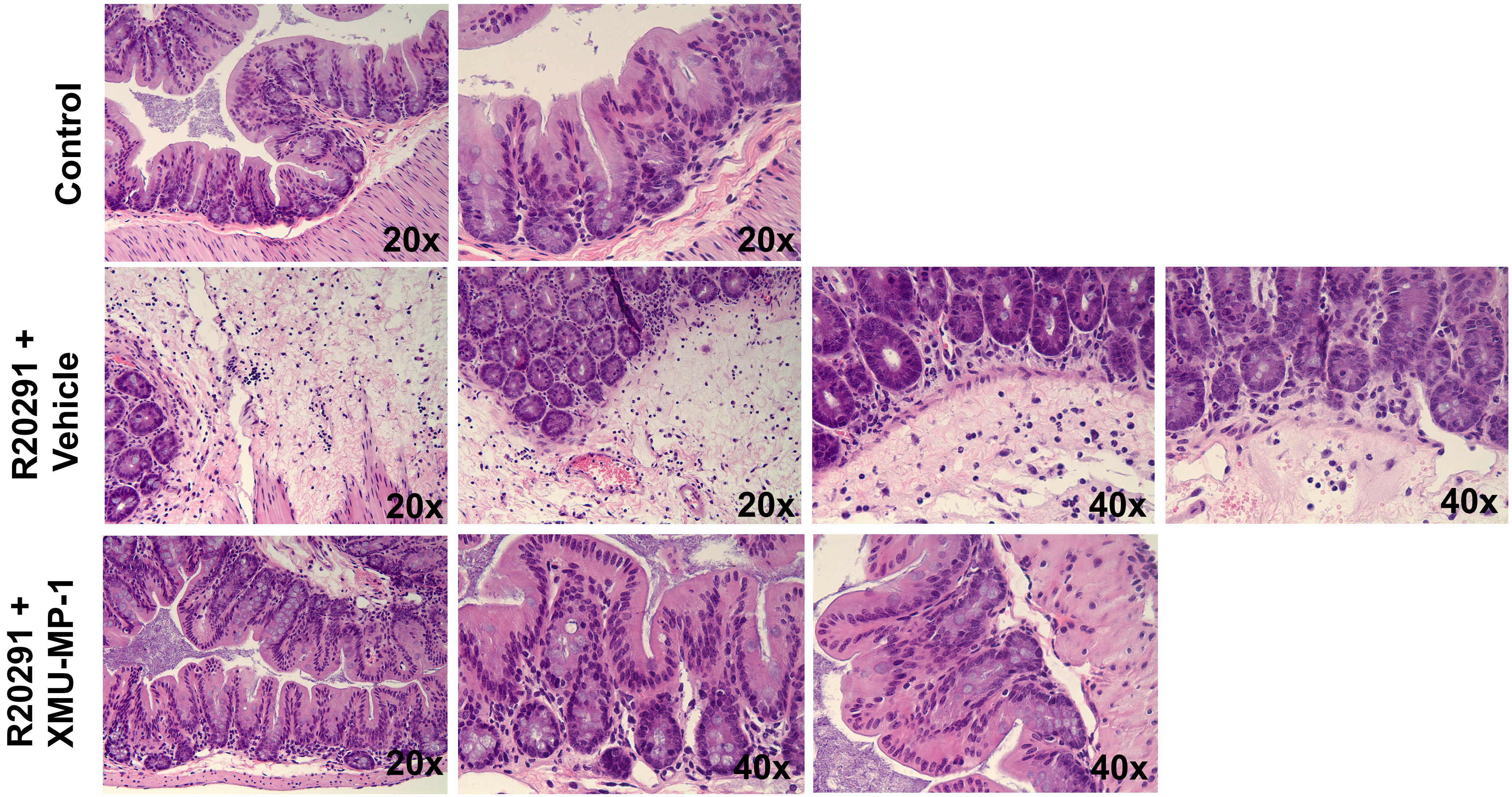

S9 Fig. Histology of ceca from C. difficile infected mice. Mice were infected with *C. difficile* strain R20291 and received daily intraperitoneal injections with 1 mg/kg of XMU-MP-1 or vehicle starting 2 days before infection and continuing through the length of the experiment. Mice were euthanized at 96 h post infection and excised ceca were H&E stained.
