# Supplementary material for: Discovery of Hippo signaling as a regulator of CSPG4 expression and as a therapeutic target for Clostridioides difficile disease
Source: PLoS Pathog. 2023 Mar 27;19(3):e1011272. doi: 10.1371/journal.ppat.1011272 (PMC10079225; doi:10.1371/journal.ppat.1011272)
Supplement: S1 Table — (PDF) [file ppat.1011272.s010.pdf]

**R20291 Growth Condition**	XMU-MP-1 600 µM	XMU-MP-1 240 µM	XMU-MP-1 120 µM	XMU-MP-1 60 µM	Vehicle (DMSO)
**OD_{600}**	0.86	0.85	0.88	0.87	0.88

S1 Table. *In vitro* growth of *C. difficile* strain R20291 in the presence and absence of XMU-MP-1.
